# Prior Infection with Torque Teno Virus Mitigates Influenza Pathology in Mice

**DOI:** 10.3390/v18030357

**Published:** 2026-03-15

**Authors:** Md-Tariqul Islam, Brett Webb, Sheela Ramamoorthy

**Affiliations:** 1 Department of Animal Sciences, North Dakota State University, Fargo, ND 58102, USA; mdtariqul.islam1@ndsu.edu; 2 Veterinary Diagnostic Laboratory, North Dakota State University, Fargo, ND 58102, USA; brett.webb@ndsu.edu

**Keywords:** influenza, torque teno virus, mice, pathology, ELISA, replication, coinfection

## Abstract

Respiratory infections caused by influenza viruses are frequently associated with coinfection by other infectious agents. Torque teno viruses (TTVs) are small DNA viruses that can function as opportunistic pathogens and are epidemiologically linked to influenza viruses as well as a broad spectrum of infectious and immune-mediated diseases. Among TTVs, swine torque teno viruses (TTSuVs) are unique in that they have been shown to act as primary pathogens. With the long-term objective of developing experimental tools to better understand inter-viral interactions, this study aimed to optimize a murine model of TTV and influenza virus coinfection. Experimental mice were inoculated with TTSuV1 on day 1 post infection (DPI 1), while phosphate-buffered saline (PBS)-treated mice served as negative controls. A subset of TTSuV1-infected mice was subsequently coinfected with the influenza A virus H1N1 (IAV) at either 12 or 27 days following TTSuV1 infection. An additional group of mice was maintained as an IAV only control. Mice infected with IAV were euthanized 72–84 h post-IAV infection, corresponding to DPI 15 and 30, respectively. Unexpectedly, gross and histopathological examination of lung tissues revealed that prior TTSuV1 infection significantly attenuated IAV-induced pathology in coinfected mice. Coinfected animals also exhibited a tendency toward reduced IAV replication in the lungs as measured by qPCR, TCID_50_ and HAs compared to mice infected with IAV alone, accompanied by lower levels of virus-specific antibodies to IAV at DPI 30 and TTSuV1 at DPI 15 respectively. At DPI 30, TTSuV1 genomic DNA levels in lung tissue and whole blood were higher in coinfected mice, suggestive of prolonged viremia in the coinfected group. Collectively, these findings establish baseline parameters for a murine TTV and influenza coinfection model and provide a foundation for future studies aimed at elucidating the molecular and immunological mechanisms underlying viral coinfections.

## 1. Introduction

Respiratory diseases involving influenza frequently occur as coinfections with other viruses or bacteria [1,2], with especially severe effects on pediatric morbidity and mortality. In addition to the negative effects on public health and animal production caused by seasonal influenza viruses and the periodic emergence of influenza viruses with pandemic potential, sequential or concurrent infection of influenza viruses with other viruses like SARS-CoV2 or RSV are well known to enhance the severity of clinical manifestations and disease burden [2,3]. Similarly, viral interference where pre-infection with one virus reduces the severity of another has also been reported [4]. However, the effects of comorbidities with opportunistic pathogens or members of the virome are not as well studied. Torque teno viruses (TTVs) are small, circular, single-stranded DNA viruses with broad, species-specific distribution which belong to the family Anelloviridae and genus Iotatorquevirus. They are considered constituents of the mammalian virome. The extensive published literature indicates that TTVs can act as opportunistic pathogens in both humans and animals, as evidenced by strong epidemiological associations with a range of infectious agents, including influenza viruses [5,6], as well as respiratory, hepatic diseases and autoimmune disorders [5,7,8,9]. More recently, TTV viral load has been shown to serve as a surrogate marker of immune suppression in patients undergoing organ transplantation or other immunosuppressive therapies [10,11,12,13].

Torque teno sus viruses (TTSuVs) are genetically diverse, colonize piglets early in life and consist of two species, TTSuV1 and TTSuV1. They are often detected as contaminants of biological products including vaccines, pork products, and the environment [14,15,16,17,18,19]. Further, TTSuV DNA is often detected in human sera, with human PBMC’s supporting TTSuV1 replication [20]. In previous studies, we determined that the baseline prevalence of TTSuV infection in healthy production pigs is approximately 52%. In contrast, 86% of pigs exhibiting severe respiratory disease tested positive for TTSuV1, with codetection of influenza A viruses and TTSuV1 in 82% of tested samples, suggesting that TTSuVs may influence the clinical outcome of respiratory coinfections [21]. Down-regulation of critical anti-influenza genes like Mx and RNAseL was noted when TTSuV1 ORF1 was over-expressed in swine macrophage cells [22,23]. Further TTSuV1 infection did not stimulate anti-viral or strong pro-inflammatory cytokine responses in these studies [22,23]. So, a clear understanding of whether TTSuV1 can modulate immune responses during coinfection with influenza requires assessment in a reproducible in vivo experimental system.

Experimental evidence supporting a primary pathogenic role for TTSuVs in pigs was provided by Krakowka et al., who demonstrated that infection of gnotobiotic piglets with TTSuV1 resulted in renal and hepatic lesions [24]. Moreover, coinfection with porcine reproductive and respiratory syndrome virus or porcine circovirus led to exacerbation of clinical disease associated with these viruses [25,26]. Despite accumulating epidemiological evidence implicating TTVs in immune modulation and disease pathogenesis, mechanistic insight into inter-viral interactions involving TTVs remains limited.

A major factor contributing to the underexplored molecular pathogenesis of TTVs has been the long-standing difficulty in propagating these viruses in vitro [27], which has also hindered the development of robust animal models. To address this gap, we developed a TTSuV1 infectious clone by cloning the viral genome (Gen Bank KT037083) amplified from a clinical case of porcine respiratory disease syndrome into a shuttle vector and then dimerized the genome to place two tandem copies of the genome in the plasmid vector [28]. We further demonstrated that trans-supplementation of the infectious clone with the replicase protein of a related, non-pathogenic, small DNA virus [porcine circovirus], enabled the rescue of high-titer TTSuV1, thereby overcoming a critical bottleneck in TTV laboratory culture [28]. For the first time, we recently demonstrated that infection of C57BL/6 mice with the rescued recombinant TTSuV1 resulted in productive infection, with viral antigen detected in lymphocytes. Infected mice exhibited an immuno-suppressed phenotype as evidenced by reduced lymphocyte proliferation in response to mitogens. TTSuV1-specific recall responses were minimal to absent throughout the duration of the study [29].

Existing knowledge regarding TTSuV1 and its effects as an opportunistic or primary pathogen has been derived from observational or molecular epidemiological studies rather than controlled experimental infection models. To address this gap, the primary objective of this study was to evaluate the impact of TTSuV1 coinfection on the replication and pathology of the influenza A/CA/2009 (H1N1) virus (hereafter referred to as influenza A virus or IAV) in mice, and to establish experimental tools to facilitate long-term investigation of immune interactions between IAV and TTVs.

## 2. Materials and Methods

### 2.1. Virus Cultures

Recombinant TTSuV1 culture was rescued as described before [20,28,30]. Briefly, the TTSuV1 infectious clone consisting of the TTSuV1 genome amplified (Accession # KT037083.1) from a clinical case of porcine respiratory disease syndrome was cloned into the pCR2.1 TA vector. A V5 tag (GKPIPNPLLGLDST) was inserted into the 3′ end of the ORF1 gene as a marker for antigenic and genetic detection. The genome was dimerized to improve transfection efficiency and avoid the need for recircularization of the viral genome for effective rescue [20,28]. The dimerized infectious clone was transfected into PK-15 cells (Porcine kidney cell line (PK-15N)) (005-TDV, National Veterinary Services Laboratory, Ames, IA, USA) as described before [22,23,28]. The rescued TTSuV1 virus was visualized by an immunofluorescence assay (IFA) using a TTSuV1-specific antibody [22]. Influenza/A/CA/2009/H1N1 (IAV) was cultured in Madin–Darby Canine Kidney (MDCK) cells as described before [31]. The virus cultures were titrated by the TCID_50_ method [32] and stored as aliquots in −80 °C until use.

### 2.2. Experimental Infection

**Figure 1:**
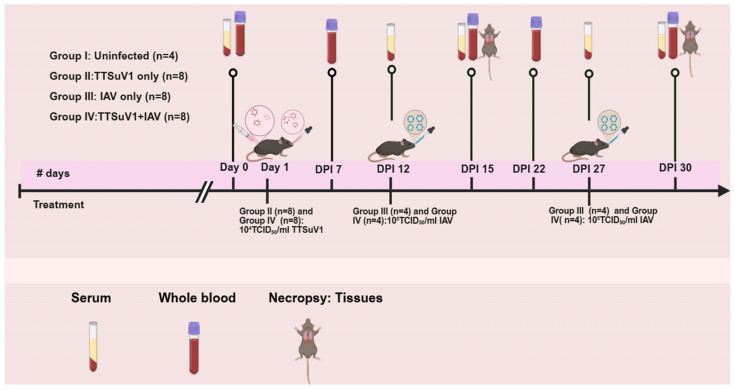
**Experimental design of the coinfection study.** Mice were divided into 4 groups: Uninfected mice (N = 4), II. TTSuV1 only (N = 8), III. IAV only (N = 8), IV. TTSuV1 + IAV (N = 8). On the day of infection (day post infection 1—DPI 1), mice in groups II and IV were infected with TTSuV1. On DPI 12 and 27, half the mice in groups III and IV were infected with IAV. Serum was collected on DPI 0, 12, 15, 27 and 30 and whole blood on DPI 0, 7, 15, 22 and 30. To evaluate the effects of coinfection on IAV pathogenesis, the IAV-infected mice were euthanized on DPI 15 and DPI 30 respectively and lungs were collected for further analysis.

The TTSuV1 and IAV coinfection study was conducted in 2–3-week-old female C57BL/6J mice (Jackson laboratory, Bar Harbor, ME, USA) [33]. Mice were acclimated for 5 days on arrival. They were housed in groups of 4 mice per cage with free access to water and standard rodent feed pellets, with a 12 h light and dark cycle. Twenty-eight mice were randomly distributed by the operators into one of four groups as follows: I. Uninfected mice (N = 4), II. TTSuV1 only (N = 8), III. IAV only (N = 8), IV. TTSuV1 + IAV (N = 8). At a confidence level of 95%, and power of 0.80, and the expectation that 100% of mice will be infected [29,34], at least 4 mice were assigned to each treatment group. Each mouse was considered a biological replicate. On the day of infection (day post infection 1 or DPI 1), mice in groups II and IV were infected with 10^4^TCID_50_/mL recombinant TTSuV1, 50 µL intranasally and 250 µL intramuscularly, to constitute a total dose of 300 µL of 10^4^TCID_50_/mL per mouse. A combination of the intramuscular and intranasal routes was selected to effectively induce respiratory and systemic infection based on prior optimization [29]. The control mice were administered the same volume of PBS by the same route. On DPI 12 and 27, mice in groups III and IV (N = 4 for each group and time point respectively) were infected with 10^5^TCID_50_/mL of IAV, 50 µL intranasally. To evaluate the effects of prior TTSuV1 infection on IAV pathogenesis, half the mice in each group were euthanized with isoflurane anesthesia followed by cervical dislocation on DPI 15 and DPI 30 respectively, at about 72–84 h post-IAV infection. The left lung was collected for qPCR (bottom half), virus isolation and quantification (top half). The entire right lung was fixed in 10% phosphate buffered formalin to evaluate IAV-induced lesions. Serum was collected on DPI 0,12, 15, 27 and 30 to assess antibody responses. Whole blood was collected on DPI 0, 7, 15, 22 and 30 for TTSuV1 qPCR (Figure 1). Mice were housed under BSL2 conditions and mouse study was carried out under the supervision of and with approval from the N. Dakota State University Institutional Animal Care and Use Committee (IACUC) under protocol number IACUC20210007. Intranasal administration of inoculum was carried out under standard inhalant isoflurane anesthesia.

### 2.3. Clinical Observations

The mice were observed daily for signs of morbidity or loss of condition. Body temperatures were measured daily for 72–84 h post-IAV infection. Clinical signs during the post-IAV infection period were scored as 1 = Mild, 2 = Moderate, 3 = Severe based on activity, inappetence, loss of condition, nasal or ocular discharge, huddling and ruffled coats. Possible conditions for exclusion from the study included random physical abnormality such as malocclusion, limb dragging, obvious tumors, infected scratches, or deformity or behavioral abnormality such as head tilt, running in circles, extreme agitation or aggression, vocalization, ruffling/hunching, lethargy, or shivering or loss of more than 20% body weight. However, no animals were excluded from this study.

### 2.4. Antibody Responses and Replication of IAV

#### 2.4.1. Antibody Responses to IAV

**Figure 2:**
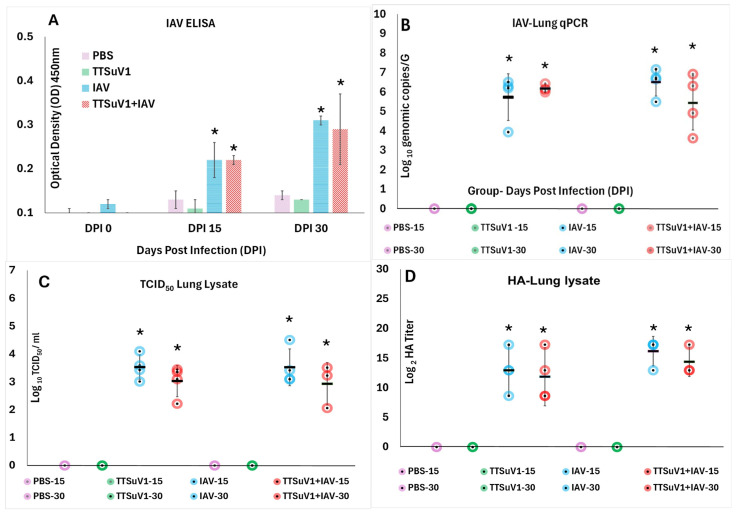
**IAV antibody responses and replication**. (**A**). **Antibody responses against IAV.** Mean anti-IAV IgG responses as measured by ELISA, Y axis—Optical density (OD). (**B**). **IAV RNA quantification in lung tissue.** Mean RNA genome copies of IAV per g lung tissue as measured by an IAV-specific qPCR, Y axis—Log_10_ genome copy numbers per g. (**C**). **Replicative IAV quantification by TCID_50_ titration.** Mean IAV TCID_50_/mL lung lysate, Y axis—Log_10_ TCID_50_/mL (**D**). **Replicative IAV quantification by HA titration.** Mean IAV HA titers from lung lysate, Y axis—Log_2_ HA titers/mL. X axis—Days post infection (DPI); Treatments: Magenta—PBS, Green—TTSuV1, Blue—IAV, Red—TTSuV1 + IAV. Individual values are represented by circles, the mean by horizontal, black bars and error bars represent the standard deviation. * *p* ≤ 0.05 when compared pairwise to the uninfected control group, Student’s *t*-test. The pairwise comparison between the IAV and TTSuV1 + IAV groups was not statistically significant as *p* was ≥0.05.

To measure IAV-specific IgG responses by ELISA, a recombinant IAV H1N1 HA antigen (BEI resources, NR-51668) was used as the capture antigen at a 1:5000 dilution, essentially as described before [35,36]. A 1:100 dilution of the test sera was evaluated in duplicate after a blocking step (General Block, Immunochemistry technologies Inc., Bloomington, MN, USA). Detection was achieved with goat anti-mouse IgG HRPO-conjugate at a 1:5000 dilution (KPL, Milford, MA, USA), followed by TMB substrate (KPL, Milford, MA, USA). Positive and negative controls were incorporated for each assay (Figure 2A).

#### 2.4.2. Quantification of IAV RNA in Lungs by qPCR

IAV RNA in lungs was quantified as described before [31,36]. Total nucleic acids were extracted from 50 mg lung tissue collected from the lower left lung lobes (MagMAX total nucleic acid isolation kit, Thermo Scientific, Waltham, MA, USA). The extracted RNA was tested using a commercial one-step quantitative reverse transcription polymerase chain reaction (qRT-PCR) kit, and the Path-ID RT-PCR Kit (Thermo Scientific, Waltham, MA, USA) targeting the matrix gene. The manufacturer’s instructions (Ref. [37]) and the standard operating procedures of the North Dakota State University Veterinary Diagnostic Laboratory [31,36] were followed. Serial log dilutions of the IAV culture prepared as described above were used to generate a standard curve. No-template controls were included in all assays (Figure 2B).

#### 2.4.3. IAV Isolation and Quantification

To measure replicative IAV in lung tissue, lung lysates were prepared as described before [29]. Briefly, 50 mg of lung tissue from each mouse in each group was macerated and filtered through a 0.22 μm filter. The lysates were centrifuged at low speed for 5 min for each group. A total of 100 uL of lysate from each mouse was layered on pre-formed MDCK monolayers and incubated for 72 h for virus isolation. The isolated virus cultures were stored at −80 °C until further testing by a standard TCID_50_ assay [31,32,36] (Figure 2C).

#### 2.4.4. IAV Quantification by the Hemagglutination Assay (HA)

Lung lysates, prepared as described above, were subjected to a HA using standard protocols [31,36,38]. Briefly, serial doubling dilutions of each lysate were mixed with equal volumes of 0.5% chicken RBCs (Lampire Biological Laboratories, Pipersville, PA, USA), mixed gently, and incubated for 15 min. To record the HA titers, wells with hemagglutination were scored as positive while wells with button formations of RBCs were scored as negative. Mean Log_2_ transformed values were used for analysis (Figure 2D).

#### 2.4.5. Lung Pathology

Microscopic lung lesions induced by IAV infection were evaluated in a blinded manner by a board-certified veterinary pathologist. The evaluation was carried out using a previously described scoring system [39]. Some modifications were incorporated to ensure a detailed examination of the lung, bronchi and alveoli. For the lung, the amount of lung affected, perivascular and peribronchiolar lymphoid hyperplasia, and vasculitis/vessel injury were evaluated. Epithelial attenuation, inflammation and intraluminal debris were scored for the bronchi and bronchioles. Alveolar inflammation, histiocytosis, necrosis/fibrin, edema, septal cellularity, type II pneumocyte hyperplasia, and interstitial fibrosis were scored for the alveoli. The scoring scale was as follows: 0 = no significant change; 1 = minimal: scattered cell necrosis, vacuolation or inflammation affecting 5 to 10% of the tissue section; 2 = mild: scattered cell necrosis, vacuolation or multifocal inflammation with few inflammatory cells affecting 10 to 25% of the tissue section; 3 = moderate: multifocal vacuolation or sloughed, necrotic cells or thin layer of cells (<5 cell layer thick) affecting 25 to 50% of the tissue section; 4 = marked: multifocal/segmental necrosis, epithelial loss or effacement or thick layer of cells (>5 cell layer thick) affecting 50 to 75% of the tissue section; 5 = severe: coalescing areas of necrosis, parenchymal effacement and confluent areas of inflammation in >75% of the tissue section. The total scores for the 4 mice in each respiratory compartment, group and time point are presented in Table 1.

### 2.5. TTSuV1 Antibody Responses, Replication and Pathology

#### 2.5.1. Antibody Responses Against TTSuV1

**Figure 3:**
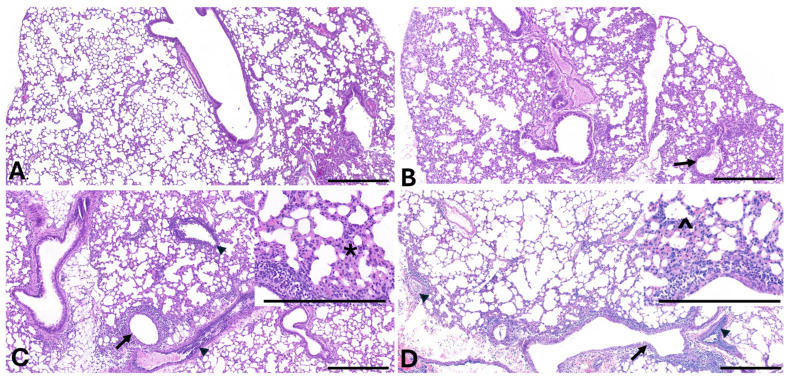
**IAV lung lesions:** Representative photomicrographs depicting the constellation of changes observed in treatment groups (B-TTSuV1 only, C-IAV only, D-TTSuV1 + IAV) compared to uninfected control (**A**). Observed lesions were restricted to grade 1–3 (**B**–**D**, respectively) with grade 4 and 5 lesions not observed in any animals. Variable degrees of epithelial attenuation to erosion of the airway epithelium, with accumulations of intra-luminal debris (arrows—primarily in (**C**,**D**)) and variable degrees of perivascular inflammation to vasculitis (arrow heads). Increased alveolar septal cellularity (*) can also be observed in (**B**–**D**) (detailed in (**C**,**D**) insets) with some alveoli containing edema and low numbers of neutrophils and histiocytes (^). Scale bar = 200 µm, hematoxylin- and eosin-stained sections.

Anti-TTSuV1 IgG responses were measured by an ORF2-specific ELISA as described previously [29]. Briefly, a 1:100,000 dilution of recombinant TTSuV1 ORF2 antigen was coated on 96 well ELISA plates (High Bind Microplate, Corning^®^, Corning, NY, USA) in coating buffer. Following a blocking step (General Block, Immunochemistry technologies Inc., Bloomington, MN, USA), the sera were tested at a 1:50 dilution in duplicate. Detection was achieved with goat anti-mouse IgG HRPO-conjugate at a 1:5000 dilution (KPL, Milford, MA, USA), followed by TMB substrate (KPL, Milford, MA, USA). Positive and negative controls were incorporated for each assay (Figure 3A).

#### 2.5.2. TTSuV1 qPCR

Copy numbers of TTSuV1 DNA in whole blood and lung was quantified by a TTSuV1-specific qPCR as previously described [28,29]. Briefly, DNA was extracted using commercial kits, the QIAamp Blood Mini easy Kit and QIAamp DNA tissue Kit (Qiagen, Valencia, CA, USA). The QuantiFast Probe PCR mix (Qiagen, Valencia, CA, USA) was mixed with 0.4 µM of the (5′-TACCCGGCTTTGCTTCGACAGTG-3′) and (5′-GCCATAGATTTCTAGCGATCCCAATTGCG-3′) primers each, 2 µM probe 5′-FAM/CACACAACACAGCAGGAA/3IABkFQ-3′, and 10 µL of extracted DNA. The PCR reaction was carried out under standard conditions and an annealing temperature of 57 °C for 30 s. Plasmid DNA containing the cloned TTSuV1 genome was used to generate a standard curve. A no-template control was included in each run.

#### 2.5.3. TTSuv1 Lymphoid Lesions

**Figure 4:**
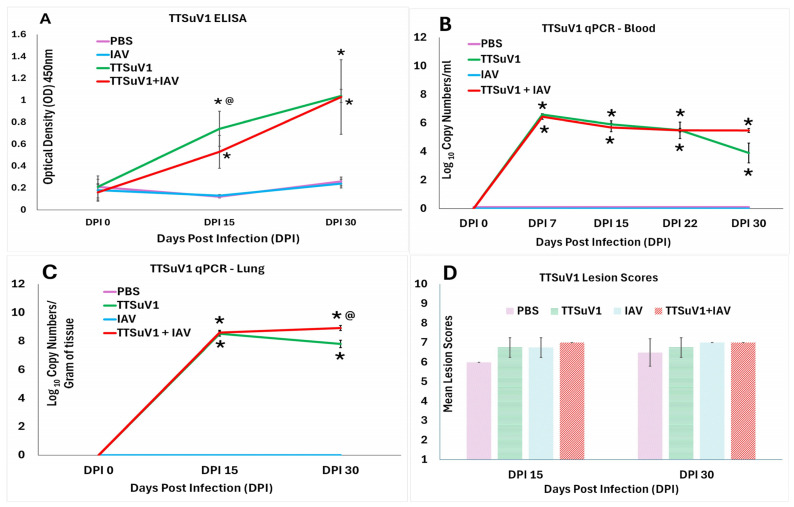
**TTSuV1 serological responses, replication and lesions.** (**A**). **Antibody responses against TTSuV1.** Mean anti-TTSuV1 IgG responses as measured by an ORF2 antigen-specific ELISA. Y axis—Optical density (OD). (**B**). TTSuV1 genomic DNA in whole blood. (**C**). **TTSuV1 genomic DNA in lung**. Copy numbers of TTSuV1 genomic DNA as measured by a TTSuV1-specific qPCR. Y axis—Log_10_ copy numbers per mL or g respectively. (**D**). **TTSuV1-induced pathology.** Y axis—mean lymphoid lesion scores. Scoring system: marked hypoplasia = 1, moderate hypoplasia = 2, mild hypoplasia = 3, normal = 4, mild hyperplasia = 5, moderate hyperplasia = 6, marked hyperplasia = 7. Extramedullary hematopoiesis was scored as follows: normal = 1, mild = 2, moderate = 3, marked = 4. The mean of the cumulative score for cellularity and extramedullary hematopoiesis is represented. X axis—Days post infection (DPI); Treatments: Mauve—PBS, Blue—IAV only, Green—TTSuV1 only, Red—TTSuV1 + IAV. * *p* ≤ 0.05 when compared to the uninfected control group, @ *p* ≤ 0.05 when compared to the TTSuV1 only control group, Student’s *t*-test.

As we previously found that TTSuV1 infection only induced mild microscopic lymphoid changes [29] in infected mice, spleen tissue was fixed in 10% formaldehyde, stained with hematoxylin and eosin and examined by a board-certified veterinary pathologist in a blinded fashion. The scoring system used was as described before [29,40] and as follows: marked hypoplasia = 1, moderate hypoplasia = 2, mild hypoplasia = 3, normal = 4, mild hyperplasia = 5, moderate hyperplasia = 6, marked hyperplasia = 7. Extramedullary hematopoiesis was scored as follows: normal = 1, mild = 2, moderate = 3, marked = 4 (Figure 4D).

#### 2.5.4. Data Analysis

Statistical analysis was performed using Microsoft Excel 365 or Minitab software version 22.3. TTSuV1 and AIV-specific Ab responses, copy numbers, pathology scores, TCID_50_, and HA titer qPCR data were assessed for normal distribution and analyzed by the parametric Student’s *t*-test. Differences between groups were considered significant at the level of *p* < 0.05. While the figures and graphs depict mean values, the tables include the total scores for each respiratory compartment for the 4 mice/group. Standard deviations and statistical significance for differences between test groups are also presented in the figures and tables.

## 3. Results

### 3.1. Clinical Signs

Observation of mice for clinical signs of lethargy, loss of condition, and respiratory distress post-IAV infection did not reveal significant changes during the post-IAV infection period. Similarly, there were no significant differences in body temperatures between the pre- and post-IAV exposure days for each group or between groups.

### 3.2. IAV Antibody Responses and Replication

#### 3.2.1. Coinfection Does Not Affect Early IAV-Specific Antibody Responses

An IAV-specific ELISA was used to detect antibody responses to IAV. As the sample was collected 72–84 h post-IAV infection, before robust antibody responses could be developed, low levels of anti-IAV IgG antibodies were detected at both the DPI 15 and 30 time points. The overall response was slightly stronger at DPI 30 when compared to DPI 15, likely as the mice were older. The IAV-specific IgG levels were lower at DPI 30 in the dually infected mice. However, there were no statistically significant differences between the single and dual infection groups at both time points. As expected, the mice in control PBS and TTSuV1 only groups and the pre-IAV infection samples collected at DPI 12 and 27 ( were serologically negative for IAV (Figure 2A) as only very low-level, non-specific reactivity was detected in these groups.

#### 3.2.2. Coinfection Tends to Reduce IAV Replication in Lungs

Robust IAV viral RNA loads were detected in lung tissue of infected mice by an IAV-specific RT-qPCR, both at DPI 15 and 30. The values were significantly different from the control PBS and TTSuV1 only groups (Figure 2B). While the differences in genome copy numbers between the IAV only and IAV plus TTSuV1 groups were not statistically significant, the trend in the dually infected mice was downward at the DPI 30 time point, indicating a possible change in replication dynamics (Figure 2B). As the detection of RNA alone does not confirm the presence of viable virus, replicative IAV in lung tissue was also quantified by the TCID_50_ (Figure 2C) and HAs (Figure 2D). Congruent with the qRT-PCR data, viral loads in the dually infected group were lower than those of mice infected with IAV alone, at both time points. However, the differences between the groups and time points were not statistically significant (Figure 2C,D). As expected, the values were statistically significantly different from the PBS and TTSuV1 control groups. IAV was not detected in the PBS and TTSuV1 only groups for the duration of the study.

#### 3.2.3. Coinfection Reduces IAV-Induced Lung Lesion Severity

When lung tissue was examined for gross and microscopic IAV-induced lesions, the coinfected mice had significantly lower lesion scores at both the DPI 15 and 30 time points when compared to mice infected with IAV alone. The lesion scores for the coinfected mice were consistently lower for all three compartments examined, i.e., lungs, bronchi and alveoli (Table 1). Lesion scores for the singly infected mice were higher at DPI 30 when compared to DPI 15. The difference was not as high between time points for the coinfected mice. As expected, IAV-induced lung pathology was not evident in mice in the control PBS and TTSuV1 only groups [29].

### 3.3. TTSuV1 Antibody Responses, Replication and Pathological Lesions

#### 3.3.1. Early TTSuV1-Specific Antibody Responses Are Lower in Coinfected Mice

Detection of TTSuV1-specific IgG responses by an ELISA showed robust antibody responses were mounted by DPI 15 and continued to increase until DPI 30. At DPI 15, the mean responses were higher in mice infected with TTSuV1 alone compared to the dually infected mice. However, by DPI 30, there was no difference between the single and dual infection groups. The mice in the PBS and IAV only groups remained negative for TTSuV1 antibodies throughout the study (Figure 4A).

#### 3.3.2. Coinfected Mice Trend Towards Higher Viral DNA Loads at DPI 30

Measurement of TTSuV1 genome copy numbers in whole blood and lung tissue using a TTSuV1-specific qPCR showed that viral DNA copy numbers peaked significantly by DPI 7 in both the single and dual infection groups. However, the TTSuV1 genomic DNA copy numbers started declining after DPI 15 in the singly infected mice. At DPI 30, TTSuV1 DNA was not detected in 1/4 mice in the single infection group and 2/4 mice in the dual infection groups. Despite non-detection in some individuals, the mean load was found to be significantly higher in the dual infection group at DPI 30. As expected, the mice in the PBS and IAV only control groups remained negative for the duration of the study (Figure 4B,C).

#### 3.3.3. Coinfection Does Not Increase TTSuV1 Lymphoid Lesion Scores

As previously noted, TTSuV1 infection resulted in mild lymphoid hyperplasia in the treated mice. However, the lesion scores were not significantly different from the control mice administered PBS or IAV alone, or from the coinfected mice. While all infected mice had higher lymphoid lesion scores when compared to the PBS control group at DPI 15, the differences were not statistically significant (Figure 4D).

## 4. Discussion

The extensive genetic diversity, broad host range, and ubiquitous distribution of torque teno viruses (TTVs) suggest the evolution of highly sophisticated mechanisms that enable long-term coexistence with the host immune system [41]. We and others have demonstrated that TTVs infect and replicate in lymphocytes, leading to functional immune modulation during early infection [22,23,29,42,43,44]. TTVs have been reported to engage Toll-like receptor 9 and induce pro-inflammatory cytokine responses [45]. However, they have also been shown to impair NF-κB signaling [46]. More recent studies demonstrate that TTV infection promotes CD8^+^ T cell exhaustion and upregulates inhibitory receptors on natural killer cells [42]. Collectively, these findings indicate that TTV infection is associated with diverse and, in some cases, opposing immunological effects. Accordingly, the well-documented epidemiological association of TTVs with a broad range of disease conditions is not unexpected [8,47,48].

As one of the most abundant constituents of the mammalian virome, a detailed understanding of TTV immunobiology and its contribution to health and disease is essential. However, progress in this area has been constrained by the long-standing inability to culture TTVs in vitro and the lack of robust in vivo models [27]. Our recent description of a mouse model of TTSuV1 infection provides a framework for examining viral interactions under controlled experimental conditions [29]. Extending this work, the primary contribution of the present study is the establishment of the first reproducible rodent model of TTV/influenza virus coinfection, providing a platform for more in-depth investigations into TTV biology and host interactions.

Consistent with previous reports, infection with the 2009 H1N1 influenza A virus (IAV) resulted in efficient viral replication, induction of lung pathology, and modulation of immune mediators without causing overt clinical disease in mice [34,49,50,51]. We have also previously shown that TTSuV1 establishes productive infection in mice without inducing observable clinical signs [29], which explains the absence of overt disease in any experimental group in the current study. It is also important to note that controlled experimental infection models do not fully recapitulate complex field conditions, where environmental factors, coinfections, and metabolic comorbidities can substantially influence disease outcomes.

Based on prior studies demonstrating that peak acute IAV replication and lung pathology occur at approximately 3–4 days post-infection [34], this time window was selected for sample collection. As this early time point precedes the development of robust humoral immunity, only low-level IAV-specific IgG responses were detected (Figure 2A), precluding meaningful correlations between antibody responses and viral replication. While IAV infectivity and replication were comparable to published reports [34,50], differences in viral loads between singly infected and coinfected groups did not reach statistical significance (Figure 2B–D). To strengthen analytical rigor, lung viral loads were quantified using three independent assays. Notably, all three methods revealed a consistent trend toward lower IAV loads in coinfected mice compared with singly infected mice, particularly at DPI 30 (Figure 2B–D). However, due to the short observation period and lack of statistical significance, these findings are interpreted with caution and the direct interpretation that reduced IAV loads were responsible for the mitigation of pathology in coinfected groups is avoided. A limitation of the study is that animals were euthanized at 72–84 h post-IAV infection; a longer observation period may have allowed clearer differences to emerge in parallel with maturation of the adaptive IAV immune response.

Although not statistically significant, reduced IAV replication in coinfected mice was accompanied by decreased IAV-associated lung disease (Table 1). As reported in other studies, infection with the 2009 H1N1 virus induced mild to moderate bronchiolitis, bronchitis, peribronchiolar alveolitis, and inflammatory infiltrates (Figure 3), confirming that the IAV infection model was consistent with established literature. The observation that prior TTSuV1 infection mitigated IAV-induced disease was unexpected; however, it is well recognized that outcomes of viral coinfections are shaped by complex and multifactorial host viral interactions [52,53]. The specific factors contributing to this observation were not examined in the present study. We have previously demonstrated that TTSuV1 infection suppresses lymphocyte proliferative responses to mitogenic stimuli and viral antigens. Although a detailed mechanistic analysis was beyond the scope of this study, it is plausible that the early immunosuppressive phenotype induced by TTSuV1 [29] dampened the pro-inflammatory responses such as increased TNF-α, IL-6, IL-1β, IL-8 and chemokines associated with IAV infection [54], thereby reducing lung pathology. For example, we have previously reported that transfection of the TTSuV1 ORF1 protein in a macrophage cell line resulted in upregulation of IL-10, PD-1 and SOC-1 at early time points [22]. Additional possible mechanisms including, interference with antiviral immune signaling, altered expression of viral proteins, host transcription factors, immune mediators, cytokines, receptors, or replication factors may also contribute to the observed outcomes [52,53]. Whether such effects contributed to the observed differences in lung pathology cannot be determined from the current data but will be the focus of future investigations.

TTSuV1 infectivity and replication kinetics in the coinfection model were consistent with our previous report using a singular TTSuV1 mouse infection model, where we demonstrated viable viral replication by flow cytometry and viral isolation form tissues of infected mice, underscoring the reproducibility of the system (Figure 4A–C) [29]. As we previously found that pathological changes in TTSuV1-infected mice are limited to mild lymphoid changes [29] and neither TTSuV1 nor 2009 H1N1 IAV is known to induce significant extrapulmonary pathology in this model, only the spleens were examined for lymphoid changes associated with TTSuv1. In contrast to IAV replication patterns, TTSuV1 DNA levels in two out of four coinfected mice showed an increasing trend over time, suggesting altered viral dynamics or delayed TTSuV1 clearance in these mice. This observation may be attributable, in part, to reduced TTSuV1-specific antibody responses at DPI 15 in the coinfected group (Figure 4A) and is consistent with field observations reporting increased TTV titers during coinfections and in immunosuppressed hosts [5,25,55,56,57] and as a marker of immune suppression [13,58]. While the differences in lymphoid lesion scores were not statistically significant between the groups (Figure 4D), the additive effects of lymphopenia and lymphoid depletion induced by both viruses [29,34] may further compromise effective TTSuV1 clearance but was not directly assessed in this study.

In summary, the limitations of this study include the short post-IAV infection observation period and the absence of detailed mechanistic analyses to explain the unexpected attenuation of IAV pathology during TTSuV1 coinfection. Factors such as infection timing, order of infection, and viral/mouse strain selection, which are known to influence coinfection outcomes, were not explored but will be the focus of future studies. Additionally, collection of samples at additional intermediate or later time points could have helped to narrow down the protective effects of TTV temporally, but were not explored. As IAV is an acute virus with resolution of viral infection occurring in about a week, while TTVs establish chronic long-term infections, the reverse order of infection with IAV followed by TTV was not explored in this study. Similarly, the use of more virulent mouse-adapted IAV strains could alter the level of protection against pathology offered by prior TTV infection. Nevertheless, this work represents a critical first step toward establishing baseline parameters for a mouse model of TTV and IAV coinfection and provides an important foundation for future studies aimed at elucidating the virological and immunological roles of Anelloviruses, a major component of the mammalian virome.

## Figures and Tables

**Table 1:** Consolidated influenza lung lesion scores.

Treatment	Lung	Bronchi	Alveoli	Total
DPI 15				
PBS	0.0	0.0	0.0	0.0
TTSuV1 only	0.0	0.0	0.0	0.0
TTSuV1 + IAV	14.00 ± 1.26 *	14.00 ± 1.27 *	9.00 ± 0.55 *	37.00 ± 1.01 *
IAV only	39.00 ± 1.31	33.00 ± 1.06	27.00 ± 1.10	99.00 ± 1.40
DPI 30				
PBS	0.0	0.0	0.0	0.0
TTSuV1 only	0.0	0.0	0.0	0.0
TTSuV1 + IAV	23.00 ± 1.03 *	20.00 ± 0.89	10.00 ± 0.91	61.00 ± 1.03 ^$^*
IAV only	37.00 ± 1.01	24.00 ± 1.13	19.00 ± 0.94	80.00 ± 1.25

* *p* ≤ 0.05 TTSuV1 + IAV group when compared pairwise to the IAV only group for each respiratory compartment or total score at each time point, Student’s *t* test. ^$^ *p* ≤ 0.05 for each respiratory compartment or total score at DPI 30 when compared to the DPI 15 time point, Student’s *t* test. DPI 15-TTSuV1-infected mice were exposed to Influenza/A/CA/2009/H1N1 (IAV) on DPI 12 and euthanized on DPI 15. DPI 30-TTSuV1-infected mice were exposed to Influenza/A/CA/2009/H1N1 (IAV) on DPI 27 and euthanized on DPI 30.

## Data Availability

Data will be made available as required.

## Abbreviations

The following abbreviations are used in this manuscript:

TTV	Torque teno virus
TTSuV	Torque teno sus virus
DPI	Days post infection
PBMC	Peripheral blood mononuclear cells
TCID_50_	Tissue culture infective dose 50
qPCR	Quantitative polymerase chain reaction
PBS	Phosphate-buffered saline
h	Hour/hours
Min	Minutes
